# Bioactive Properties of Extracts from *Plectranthus barbatus* (*Coleus forskohlii*) Roots Received Using Various Extraction Methods

**DOI:** 10.3390/molecules27248986

**Published:** 2022-12-16

**Authors:** Kamila Kulbat-Warycha, Joanna Oracz, Dorota Żyżelewicz

**Affiliations:** Institute of Food Technology and Analysis, Faculty of Biotechnology and Food Sciences, Lodz University of Technology, 2/22 Stefanowskiego St., 90-537 Lodz, Poland

**Keywords:** *Plectranthus barbatus* extract, antioxidant capacity, polyphenols, flavonoids, shaking water bath extraction (SWE), ultrasound-assisted extraction (UAE), microwave-assisted extraction (MAE)

## Abstract

The aim of this study was to verify various extraction methods: shaking water bath extraction (SWA), ultrasound-assisted extraction (UAE) and microwave assisted extraction (MAE), and their parameters to optimize the extraction yield as well as maximize the concentration of polyphenols in *Plectranthus barbatus* extracts. Extracts were obtained from dried roots of *P. barbatus* in various degrees of fragmentation and analyzed for content of polyphenols, antioxidant capacity and flavonoids. Additionally, phenolic compounds in extracts were analyzed using the UHPLC–DAD–ESI–MS/MS method. The conducted research showed that roots of *P. barbatus* are rich in polyphenolic compounds. A total of 15 phenolic compounds, belonging to the group of phenolic acids and their derivatives, were identified. The extraction yield was similar for all extraction methods and averaged 31%. Irrespective of the extraction method, the yield was the lowest in the case of using 80% ethanol as the solvent. The extracts obtained from the finer fraction were characterized by a higher antioxidant capacity as well as a higher concentration of polyphenolic compounds including flavonoids. UAE seems to be the most effective method for extraction of polyphenols from *P. barbatus* roots. Regardless of the extraction method, ethanol was a better extractant than distilled water. All ethanolic extracts were characterized by a high antioxidant capacity. The 80% ethanol solution was considered the best solvent for the extraction of flavonoids, while the 40% and 60% ethanol solutions were sufficient for the effective extraction of polyphenolic compounds in general.

## 1. Introduction

*Plectranthus barbatus* Andr. (formerly known as *Coleus forskohlii*) is a member of the *Lamiaceae* family, known as the mint family. Popular herbal plants also belong to this botanical family, e.g., sage (*Salvia*), basil (*Ocimum*) and thyme (*Thymus*) [1].

Because of its medicinal properties, *P. barbatus* was used for ages in traditional Ayurvedic medicine as well as in folk medicine in African countries, Brazil and in the Far East. It is used in the herbal treatment of a variety diseases. One of the most common uses is the herb’s infusion to treat digestive system disorders [2,3,4]. This effect is supported by the fact that it stimulates the secretion of hydrochloric acid and pepsinogen in the stomach [5]. Additionally, it alleviates the symptoms associated with painful urination, and is also useful in bronchitis and asthma as well as cardiovascular disorders. In Brazil, it is called “falso boldo” and most often used in the form of decoctions and infusions of leaves as a remedy for stomach ailments, soothing inflammation, as well as a diuretic and expectorant [3,6].

The main biologically active compounds isolated from *P. barbatus* are polyphenols, diterpenes, essential oil components and alkaloids. Among the substances present in the root extracts, two main groups of diterpenoids were distinguished: abietanes and labdans. The diterpene of the labdane family forskolin is the main, biologically active compound present only in the species *Plectranthus.* Forskolin was discovered in 1974 and originally called coleonol [2,6]. As a biologically active compound, forskolin is an adenylate cyclase (AC) activator which raises the cyclic adenosine monophosphate (cAMP) level in cells [7,8,9]. The roots are the most valuable part of the plant, as they contain the highest concentration of forskolin [2,10,11].

The main target of forskolin—adenylate cyclase (AC) exists in many isoforms and is differentially expressed in different cell types. In mammalian cells, there are nine transmembrane isoforms of AC. Most of them interact with G protein-coupled receptors. Forskolin activates 8 isoforms of AC from AC1 to AC8, but not AC9 [12]. Thus, forskolin by stimulating AC, increases the level of intracellular cAMP, which regulates and influences the activity of many enzymes in cells involved in various biological processes from metabolism to gene regulation. It is especially beneficial in many diseases such as asthma, eczema, psoriasis, hypertension, obesity or even some cancers. In these diseases, a decrease in the level of intracellular cAMP is observed [6]. Forskolin, by activating AC and thus increasing the level of cAMP, prevents platelet aggregation [13] and lowers blood pressure [14,15], also intraocular pressure [16]. In addition, forskolin stimulates lipolysis in adipocytes and may be effective in reducing body fat [17,18], which was confirmed in an animal model [19,20] and also in humans [21,22]. Equally important, forskolin is effective in the inhibition of IgE-mediated histamine and leukotriene release from basophils and mast cells [23]. It is more effective than sodium cromoglycate in the prevention of asthma attacks [24] and comparable to inhaled beclomethasone [25]. Since most of the research on forskolin dates back to the 1980s and only a few date back to recent years, there is a great need for extensive clinical trials to assess its effectiveness and possible side effects.

Apart from forskolin, the newly discovered abietane diterpene is plectrabarbene, which was isolated from *P. barbatus* aerial parts together with sugiol and 11,14-dihydroxy-8,11,13-abietatrien-7-one. The ability of these compounds to bind to the active site of the enzyme acetylcholinesterase (AchE) and thus inhibit its activity suggest that they may be a potential natural drug in the treatment of Alzheimer’s disease [26]. Previously *P. barbatus* herbal tea was found to play as an inhibitor of the brain AChE in an animal model [27].

*P. barbatus* seems to also be a source of antimicrobial and cytotoxic compounds. Extracts obtained from dried leaves of *P. barbatus* have proven active against *Helicobacter pylori* and what’s equally important, cytotoxic activity against gastric adenocarcinoma cells [28]. *P. barbatus* essential oils, thanks to their antibacterial properties, can also be useful in the treatment of dermatological diseases. The ingredients of *P. barbatus* root essential oil obtained in a supercritical extraction process are highly effective against *Propionibacterium acnes*. In addition, the oil has been found to inhibit other microorganisms associated with skin infections. It inhibits the growth of *Staphylococcus aureus,* better than the popular tea tree oil used for the care of acne-prone skin, and was effective against *Staphylococcus epidermidis.* Additionally, the oil reduces the growth of *Candida albicans* yeasts responsible for candidiasis of the skin and mucous membranes [29].

Additionally, dose-dependent antibacterial and biofilm inhibitory activity was noticed against *Listeria monocytogenes, Salmonella enterica* subsp. *enterica* serovar *Typhimurium* and *Escherichia coli* [30]. This indicates that *P. barbatus* essential oils or extracts might be a potential agent in natural food preservation.

The composition of essential oils obtained from *P. barbatus* differed depending on the place and date of herb harvesting and the content of terpenes, but mono- and sesquiterpenes were predominant. The most common compounds were: α-pinene, α-cedrene, β-cadinene, β-caryophyllene, citronellal, limonene, trans-caryophyllene, β-o-cymene and α-humulene [10,31]. Barbaterpene and barbatusterol have proven to yield cytotoxic activity against neoplastic cells [32,33].

*P. barbatus* is widely used in Africa as a natural treatment for AIDS. It appears to reduce the HIV viral load and alleviate symptoms experienced by people who are infected. In 2013, evidence of the inhibitory activity of *P. barbatus* extracts against HIV was presented for the first time [34]. Due to the fact that virus replication takes place in a highly oxidized environment, the antioxidant activity of the *P. barbatus* extract was measured by determining the scavenging capacity of the 2,2-diphenyl-1-picrylhydrazyl radical (DPPH). It was found that the use of a specific solvent (ethanol or water) in the extraction process had a large influence on the result. At a concentration of 100 μg/mL, the extract inhibited the activity of HIV-1 protease at 70%. In the view of reverse transcriptase, no significant inhibitory potential was demonstrated; nevertheless, extracts contributed to a significant reduction in HIV replication and to a reduction in the concentration of pro-inflammatory cytokines [35]. Considering all the above supplementation with *P. barbatus,* in the future, it may become a beneficial solution in supporting the pharmacological treatment of AIDS.

Considering all of the above, it seems advisable to study the properties of not only forskolin itself, but also whole extracts obtained by various methods. It is also important to optimize safe and efficient extraction methods and to conduct further research on the isolation of active compounds from *P.barbatus.*

The main goal of this research is to develop an optimal method and conditions for obtaining *P. barbatus* (*Coleus forskohlii*) extracts. For high-quality extracts, both the extraction technique and its parameters are of key importance. Plant extracts are temperature sensitive, so low-temperature extraction methods have been implemented: shaking water bath extraction (SWE), ultrasound-assisted extraction (UAE) and microwave-assisted extraction (MAE). The extraction was carried out so that the temperature of the solution did not exceed 40 °C. Another important factor influencing the quality of the extract is the selection of the extraction solvent. Extraction was carried out using distilled water and various concentrations of ethanol, which is one of the most commonly used solvents due to its effectiveness and safety (FDA approved for use). It is important in the context of the potential use of the *P. barbatus* extract as a functional component of the diet. In the second stage, after obtaining the extracts, determination of the extraction yield was determined and the antioxidant properties as well as the total polyphenols and flavonoids content. Additionally, the UHPLC–DAD–ESI–MS/MS analysis of phenolic compounds was performed.

## 2. Results and Discussion

The UAE is recognized as a potentially suitable form of extraction in the food technology and pharmaceutical industry for a wide variety of herbal extracts and may provide alternatives to other methods. The mechanism of ultrasonic extraction is well known, but research is still ongoing into factors affecting the efficiency of the process, such as time and temperature, as well as the frequency and intensity of ultrasounds. The UAE method is simple, significantly shortens the process time and increases the extraction of bioactive compounds from plant material, mainly because of enlargement of the pores in the cell walls and increased permeability of cell membranes. The phenomenon of cavitation is also of great importance in ultrasound assisted extraction. Cavitation bubbles on the surface of the sample cause erosion and breakdown of the solid sample into smaller fragments. Additionally, the phenomena of macroturbulence and micro-mixing appear. Erosion increases the availability of the solvent to the sample of plant material, which leads to higher extraction efficiency and increases the solubility of the extracted compounds [36]. What is really important is that the UAE is a suitable method to extract thermolabile compounds as it allows for efficient extraction at low temperatures. UAE is considered to be a very efficient method for extracting bioactive compounds from fruits, seeds, roots, leaves and even flowers [37,38,39,40]. However, the process conditions must be carefully defined and adapted to the type of sample and compounds to be extracted.

It should be emphasized that UAE is associated with the risk of the formation of free radicals. Although the concentration of oxidative species is rather low, it is possible that they may contribute to the oxidative degradation of compounds present in the extract. As a consequence, the chemical profile of the extract may be changed, and the total antioxidant capacity may be lower [41].

Similarly to the UAE, the extraction supported by microwaves is believed to be more effective compared to conventional extraction methods, allowing for a reduction in time and solvent consumption. The efficiency of the MAE depends mainly on the kind of a sample, sample to solvent ratio and microwave power. Solvent selection is a particularly important parameter in microwave extraction. Only polar solvents can be used in this technology, such as water, acetone, ethyl acetate, methanol and ethanol. The non-polar solvent such as hexane is not heated by microwaves [42].

The MAE has been successfully used to extract anthocyanins [43], polyphenols [44] and even essential oils [45]. However, Duan and co-authors [43] noticed that although the extraction efficiency was very high, the HPLC chromatograms of the extracts revealed that some anthocyanins were slightly hydrolyzed. These results indicate that under certain conditions, microwave radiation can lead to degradation of the extracted compounds.

One of the recent research projects proves that UAE is more effective for polyphenols and total antioxidants extracted from lemon-scented tea tree leaves when compared to conventional shaking water bath extraction (SWE) [46].

As plant extracts are temperature sensitive, low-temperature extraction methods were implemented in our experiments. The UAE is a favorable solution for isolating thermolabile substances due to the possibility of using low temperatures, but in our experiments, not only UAE but also SWE and MAE were carried out at 40 °C; that is, at a temperature below protein denaturation.

Recent studies comparing the efficiency of ultrasound-assisted extraction with conventional maceration of leaves of *Bauhinia forficata* (*Fabaceae* family) have proven the superiority of ultrasonic extraction. According to the authors, ethanol was the best solvent for the extraction of polyphenolic compounds compared to n-hexane and ethyl acetate. Optimal extraction yield and concentration of polyphenols were obtained by extraction at 41 °C and the plant material to solvent ratio at 1:20 (*w*/*v*) [47].

According to Ez zoubi et al. [48], for extraction of phenolic compounds from aerial parts (leaves, stems and flowers) of *Lavandula stoechas* (*Lamiaceae* family), the optimal parameters of the UAE are ethanol as a solvent in the concentration of 40%, plant material to solvent ratio at 1:30 (*w*/*v*) and a time of extraction 32.62 min.

In our experiments, all extracts of *P. barbatus* were prepared so that the ratio of dried plant material to solvent equal 1:20 (*w*/*v*).

As shown in Table 1, extraction yields ranged from 24.41% to 36.36%, while the average for all methods was 31%. The highest yield (36.36%) was achieved by the MEA method from the coarse fraction (0.5–1.25 mm) by using water as a solvent. A similar yield (35.10%) was also obtained from the coarse fraction extracted by UAE for 15 min using water as a solvent. What is worth noting is that the lowest efficiency of the extraction process, irrespective of the method and fraction of plant material, was obtained by extraction with the use of 80% ethanol as the solvent.

**Figure 1:**
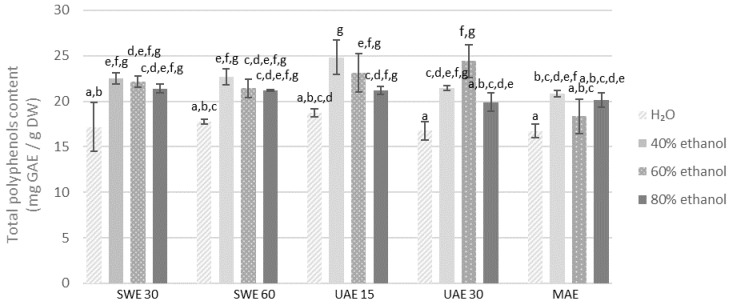
Polyphenols content in the extracts obtained from the fraction with particle size ≤0.5 mm. SWE—shaking water bath extraction; UAE—ultrasound-assisted extraction; MAE—microwave assisted extraction; 15, 30 or 60-time of extraction. Results are presented as the mean ± SD from triplicate determinations. Different letters: a, b, c, d, e, f, g indicate that samples are significantly different. The letter “a” marks the lowest value. Higher values are marked with consecutive letters of the alphabet. Bars that share the same letter within the group are not significantly different according to Tukey’s HSD test at *p* > 0.05.

**Figure 2:**
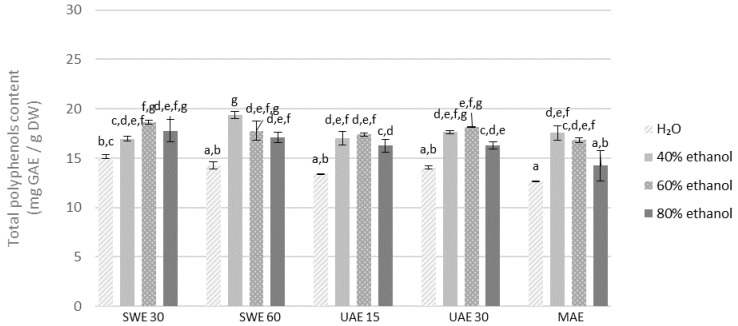
Polyphenols content in the extracts obtained from the fraction with particle size 0.5–1.25 mm. SWE—shaking water bath extraction; UAE—ultrasound-assisted extraction; MAE—microwave assisted extraction; 15, 30 or 60-time of extraction. Results are presented as the mean ± SD from triplicate determinations. Different letters: a, b, c, d, e, f, g indicate that samples are significantly different. The letter “a” marks the lowest value. Higher values are marked with consecutive letters of the alphabet. Bars that share the same letter within the group are not significantly different according to Tukey’s HSD test at *p* > 0.05.

Most likely, the decrease in the extraction efficiency may result from the denaturation of proteins present in the plant material, which blocks the release of biologically active compounds into the solution. Taking into account the concentration of polyphenols in the obtained extracts, UAE was the most efficient method. All results are given in Figure 1 and Figure 2. The highest concentration of polyphenols was recorded for the extract obtained with 40% ethanol from the fraction with particle size ≤0.5 mm during 15 min of extraction (24.81 mg GAE/g DW). Similarly, the extract obtained from the finer fraction with 60% ethanol solution during 30 min was characterized by a comparable content of polyphenols (24.44 mg GAE/g DW). The obtained results are confirmed by the literature data. The maximum extraction efficiency and the highest total concentration of polyphenols in *Lavendula stoechas* (*Lamiaceae* family) extracts required 40% ethanol concentration [48]. Similar research conducted on *Thymus serpyllum* (also *Lamiaceae*) confirmed that ethanol in the concentration of 50% was optimal for high polyphenols content in extracts obtained both by using maceration and UAE. Moreover, the extraction time did not significantly affect the concentration of polyphenols in the obtained extracts [49].

In the case of the extraction process assisted by shaking in the water bath, the highest content of polyphenols (22.66 mg GAE/g DW) was determined for the extract from the finer fraction of plant material extracted during 60 min, using 40% ethanol as a solvent. A similar value was obtained for an extraction time of 30 min with 40% ethanol (22.50 mg GAE/g DW) and 30 min with 60% ethanol (22.17 mg GAE/g DW) which may prove that extending the SWE extraction time from 30 min to 60 min does not significantly increase the amount of extracted polyphenolic compounds (*p* > 0.05).

Analysis of all the above mentioned results indicates that regardless of the method, the concentration of ethanol as a solvent is of key importance. The efficiency of the solvent used in the extraction process depends mainly on its ability to dissolve specific functional groups. The solvent can also affect the permeability of plant cell membranes and walls. Ethanol is known to increase cell membrane permeability by affecting the phospholipid bilayer [50]. The data analysis shows that the use of distilled water as a solvent resulted in obtaining extracts with the lowest total polyphenols content compared to ethanol extracts in all extraction methods. Literature data confirm that total polyphenolic compounds and antioxidant potential of herbal extracts are strongly dependent on the concentration of ethanol as a solvent. Ethanol concentration of 65% was optimal for total polyphenolic content in extracts obtained by maceration from dried rosemary leaves (belonging also to *Lamiaceae* family) [51]. Similarly, the extracts obtained from the dried root of *P. barbatus* in a Soxhlet extractor revealed that compared to other solvents, the ethanol extract contained the most bioactive compounds, with not only polyphenols but also alkaloids, tannins, saponins, carbohydrates, glycosides, terpenoids, quinones and steroids [52].

Apart from the solvent, the fragmentation of plant material is of great importance in the extraction, which was confirmed by our studies. The analysis of the results proves that, in general, higher concentrations of polyphenols in the extracts were obtained from the finer fraction (≤0.5 mm) compared to the coarser one (0.5–1.25 mm). Longer extraction time did not result in a higher concentration of polyphenols in the obtained extracts.

According to Platzer et al. [53], the Folin–Ciocalteu assay does not only determine total polyphenolic content of a sample but the reducing capacity, as its results mostly depend on the number of OH groups present in diverse substances, not only in polyphenols. In order to comprehensively assess the antioxidant properties of extracts, it is necessary to use various methods of antioxidant determination, which are based on different mechanisms. In this work, not only polyphenolic content was determined, but also flavonoids content as well as DPPH radical scavenging activity and antioxidant capacity measured by the FRAP assay.

**Figure 3:**
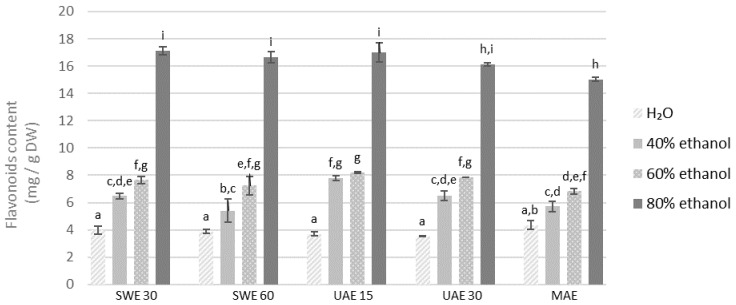
Flavonoids content in the extracts obtained from the fraction with particle size ≤0.5 mm. SWE—shaking water bath extraction; UAE—ultrasound-assisted extraction; MAE—microwave assisted extraction; 15, 30 or 60-time of extraction. Results are presented as the mean ± SD from triplicate determinations. Different letters: a, b, c, d, e, f, g, h, i indicate that samples are significantly different. The letter “a” marks the lowest value. Higher values are marked with consecutive letters of the alphabet. Bars that share the same letter within the group are not significantly different according to Tukey’s HSD test at *p* > 0.05.

**Figure 4:**
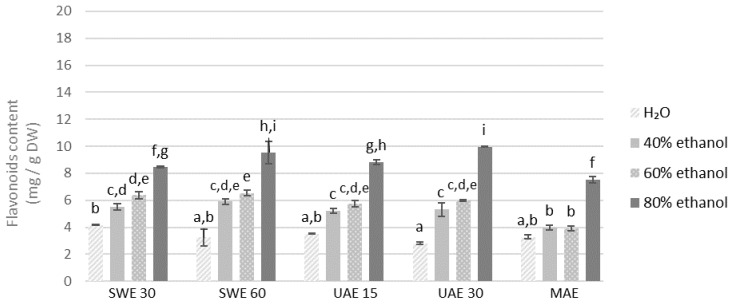
Flavonoids content in the extracts obtained from the fraction with particle size 0.5–1.25 mm. SWE—shaking water bath extraction; UAE—ultrasound-assisted extraction; MAE—microwave assisted extraction; 15, 30 or 60-time of extraction. Results are presented as the mean ± SD from triplicate determinations. Different letters: a, b, c, d, e, f, g, h, i indicate that samples are significantly different. The letter “a” marks the lowest value. Higher values are marked with consecutive letters of the alphabet. Bars that share the same letter within the group are not significantly different according to Tukey’s HSD test at *p* > 0.05.

Similar to the previously analyzed polyphenols, the same trend was visible in the case of flavonoids (Figure 3 and Figure 4), one of the main groups of polyphenols. The results showed that distilled water used as a solvent yielded the lowest quantity of flavonoids in all extraction methods. Again, similarly to polyphenolic compounds, higher concentration of flavonoids was obtained from the finer fraction. It is difficult to indicate the most effective extraction method and no increase in the concentration of flavonoids was observed as a result of a longer extraction time. The concentration of flavonoids in the obtained extracts was the lowest in the aqueous extracts and increased with the concentration of ethanol as a solvent. All samples extracted by 80% ethanol demonstrated higher levels of flavonoids when compared to other solvents.

**Figure 5:**
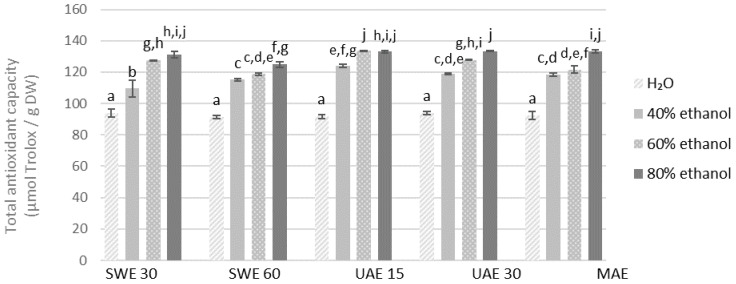
Antioxidant capacity of the extracts obtained from the fraction with particle size ≤0.5 mm determined by the method with DPPH radicals. SWE—shaking water bath extraction; UAE—ultrasound-assisted extraction; MAE—microwave assisted extraction; 15, 30 or 60-time of extraction. Results are presented as the mean ± SD from triplicate determinations. Different letters: a, b, c, d, e, f, g, h, i, j indicate that samples are significantly different. The letter “a” marks the lowest value. Higher values are marked with consecutive letters of the alphabet. Bars that share the same letter within the group are not significantly different according to Tukey’s HSD test at *p* > 0.05.

**Figure 6:**
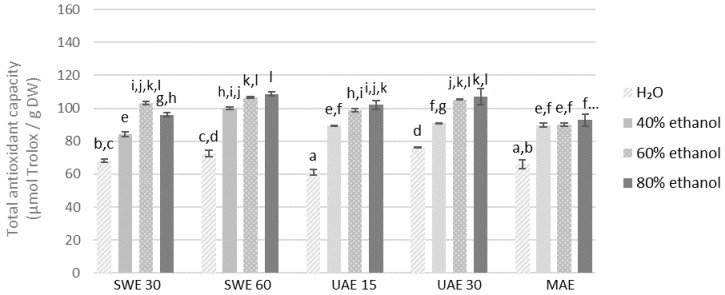
Antioxidant capacity of the extracts obtained from the fraction with particle size 0.5–1.25 mm determined by the method with DPPH radicals. SWE—shaking water bath extraction; UAE—ultrasound-assisted extraction; MAE—microwave assisted extraction; 15, 30 or 60-time of extraction. Results are presented as the mean ± SD from triplicate determinations. Different letters: a, b, c, d, e, f, g, h, i, j, k, l indicate that samples are significantly different. The letter “a” marks the lowest value. Higher values are marked with consecutive letters of the alphabet. Bars that share the same letter within the group are not significantly different according to Tukey’s HSD test at *p* > 0.05.

The highest DPPH scavenging capacity was recorded for the 60% ethanolic extract obtained from the finer fraction by UAE in 15 min (133.47 μmol of Trolox/g DW) and in 80% ethanolic extract obtained also by UAE in 30 min (133.44 μmol of Trolox/g DW), while the least for the aqueous extract obtained by the same method but from the coarse fraction in 15 min (61.15 μmol of Trolox/g DW) (Figure 5 and Figure 6).

**Figure 7:**
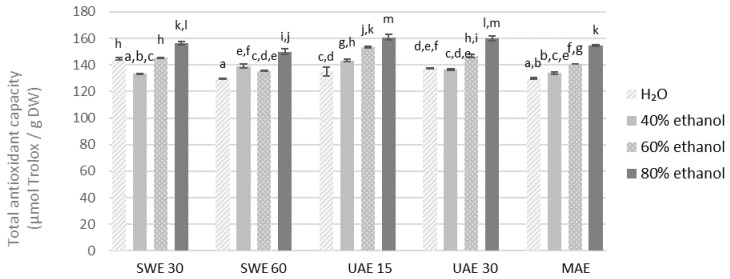
Antioxidant capacity of the extracts obtained from the fraction with particle size ≤0.5 mm determined by the FRAP method. SWE—shaking water bath extraction; UAE—ultrasound-assisted extraction; MAE—microwave assisted extraction; 15, 30 or 60-time of extraction. Results are presented as the mean ± SD from triplicate determinations. Different letters: a, b, c, d, e, f, g, h, i, j, k, l, m indicate that samples are significantly different. The letter “a” marks the lowest value. Higher values are marked with consecutive letters of the alphabet. Bars that share the same letter within the group are not significantly different according to Tukey’s HSD test at *p* > 0.05.

**Figure 8:**
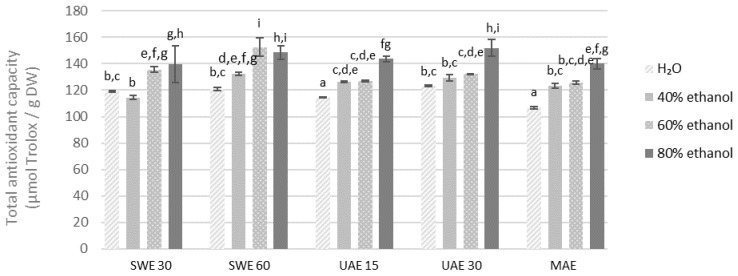
Antioxidant capacity of the extracts obtained from the fraction with particle size 0.5–1.25 mm determined by the FRAP method. SWE—shaking water bath extraction; UAE—ultrasound-assisted extraction; MAE—microwave assisted extraction; 15, 30 or 60-time of extraction. Results are presented as the mean ± SD from triplicate determinations. Different letters: a, b, c, d, e, f, g, h, i indicate that samples are significantly different. The letter “a” marks the lowest value. Higher values are marked with consecutive letters of the alphabet. Bars that share the same letter within the group are not significantly different according to Tukey’s HSD test at *p* > 0.05.

Maximum antioxidant capacity measured by the FRAP was recorded for 80% ethanolic extract obtained from the finer fraction by UAE in 15 min (160.80 μmol of Trolox/g DW) (Figure 7 and Figure 8). Regardless of the method of measuring antioxidant activity, the aqueous solutions had the lowest antioxidant capacity in all the cases. Noteworthy, antioxidant activity tended to increase with the concentration of ethanol as the extractant.

In extracts obtained with different methods from *P. barbatus* roots, based on peak retention times, UV–vis spectra and signal analysis in high-resolution mass spectra (ESI–MS and ESI–MS/MS), phenolic compounds belonging to the group of hydroxybenzoic acids, hydroxycinnamic acids and their derivatives were identified, after comparison with standards and literature data [41]. In extracts obtained from *P. barbatus* roots, a total 15 phenolic compounds, belonging to the group of phenolic acids and their derivatives, including quinic acid, potocatechuic acid, 4-hydroxybenzoic acid, 2-hydroxybenzoic acid, protocatechuic aldehyde, gentisic acid, 4-hydroxybenzoic acid *O*-hexoside, vanillic acid, caffeic acid, syringic acid, p-coumaric acid, ferulic acid, ellagic acid, hydroxygallic acid and rosmarinic acid were identified by UHPLC–DAD–ESI–MS/MS analysis (Table 2). The total amount of identified phenolics, calculated as the sum of individual compounds, in extracts ranged from 1.437 to 3.386 mg/g DW. The highest concentration of phenolic compounds was observed for the extract obtained with 40% ethanol from the fraction with particle size 0.5–1.25 mm during 60 min of SWE extraction. The lowest total phenolic content was characterized by extract from the fraction with particle size ≤0.5 mm during 30 min of SWE extraction. Rosmarinic acid (RA) was the dominant phenolic compound in almost all the extracts tested (except those extracted with water), followed by quinic acid, potocatechuic acid, caffeic acid, 4-hydroxybenzoic acid, protocatechuic aldehyde, gentisic acid and hydroxygallic acid. What is worth noting, the content of dominant rosmarinic acid was on average 5 times higher in ethanol extracts compared to aqueous extracts. Similarly, studies on another species of the genus *Plectranthus*-*P. amboinicus* showed that rosmarinic acid was the dominant bioactive compound present in high concentration also in methanol extracts [54].

Rosmarinic acid is a phenolic acid that is particularly abundant in plants of the *Lamiaceae* family. The research conducted by Benedec et al. (2015) [55] showed that the content of rosmarinic acid in ethanolic extracts of six *Lamiaceae* species ranges from 1.33 mg/g in *Rosmarinus officinalis* to 12.40 mg/g in *Origanum vulgare*. Interestingly, a positive linear relationship was observed between the antioxidant activity and the content of rosmarinic acid in the analyzed extracts. Such a relationship may indicate that rosmarinic acid is the main polyphenol responsible for high antioxidant activity of extracts.

Depending on the extraction method and fraction, the concentration of rosmarinic acid, quinic acid and potocatechuic acid ranged from 0.132 to 1.264 mg/g DW, from 0.259 to 0.980 mg/g DW, and from 0.262 to 0.543 mg/g DW, respectively. Caffeic acid, 4-hydroxybenzoic acid, protocatechuic aldehyde and gentisic acid contents ranging from 0.136 to 0.317 mg/g DW, from 0.122 to 0.246 mg/g DW, from 0.073 to 0.153 mg/g DW and from 0.067 to 0.135 mg/g DW, respectively, were observed. Other identified phenolic compounds were also determined in almost all the extracts tested, but in very low amounts. The levels of other phenolic compounds varied from trace to 0.178 mg/g DW.

## 3. Material and Methods

### 3.1. Plant Material and Chemicals

The material for the research was *P. barbatus* (*Coleus forskohlii*) roots which were purchased from Nanga (Blękwit, Poland). Before the extraction, the plant material was cut, convectionally dried at 40 °C, ground in a grinder with a cooling water jacket IKA M20 (IKA-Werke GmbH & Co. KG, Staufen, Germany) and sieved in order to isolate two fractions ≤0.5 mm and 0.5–1.25 mm. Before extraction, samples of both fractions were dried with a RADWAG MAC 50/1 moisture analyzer (Radwag, Radom, Poland) in order to determine moisture content, which is necessary to determine dry mass content in the fractions.

### 3.2. Plectranthus Barbatus (Coleus forskohlii) Root Extraction

An amount of 1 g of dry plant material was suspended in 20 mL of a solvent (distilled water or ethanol in aqueous solution in the concentration of 40%, 60% and 80%). For all methods, the solid/solvent ratio was the same 1/20 (*w*/*v*). Shaking water bath extraction (SWE) was performed by using rotator Multi Bio RS-24, (Biosan, Riga, Latvia) at 40 °C for 30 and 60 min. Ultrasound-assisted extraction (UAE) was carried out in an ultrasonic water bath Elmasonic S 100H (Elma Schmidbauer GmbH, Singen, Germany) with a generator frequency of 37 kHz (150 W) at temperature of 40 °C for 15 and 30 min. The microwave-assisted extraction (MAE) was performed using a Gourment 8601 microwave-convection oven (Bosch-Siemens Hausgeräte GmbH, Gerlingen-Schillerchoche, Germany) with a microwave power of 360 W. The extraction time was selected experimentally so that the temperature of the solution at the end of the process was 40 °C: 10 s for distilled water as a solvent, 9 s for a 40% ethanol solution, 8 s for a 60% ethanol solution and 7 s for an 80% ethanol. All variants of the experiment with parameters are presented in Table 3.

The solutions were then centrifuged at 6000 rpm (4800 G) for 10 min using a Centurion Scientific K2015R centrifuge (Stoughton, United Kingdom). Subsequently, 5 mL of each supernatant was poured into pre-weighed 50 mL falcon tubes to determine the extraction yield. The remainder of the extracts was used for the analyses.

### 3.3. Determination of the Extraction Yield

The supernatants were evaporated to dryness at 40 °C. The extraction efficiency was calculated as the quotient of the mass of the dried extract to the mass of the plant material subjected to the extraction process and expressed as a percentage.

### 3.4. Determination of Total Antioxidant Capacity

Two analytical techniques were employed to determine antioxidant capacity of the extracts. Determination of total antioxidant capacity using the ability of antioxidants to reduce the DPPH radicals was performed according to the method described by Oracz & Żyżelewicz in 2020 [56] and expressed as μmol of Trolox equivalent per 100 g dry weight of extract (μmol TE/100 g DW). Total antioxidant capacity was also tested by the FRAP (Ferric Reducing Antioxidant Power) assay described in detail by Georgiadou et al. in 2018 [57]. 1.98 mL of freshly prepared FRAP solution (0.3 M acetate buffer (pH = 3.6) containing 10 mmol 2,4,6- tripyridyl-1,3,5-triazine (TPTZ) dissolved in 40 mM HCl and 40 mmol FeCl_3_ × 10H_2_O) was added to 15 µL of each extract and incubated at 37 °C in the dark. The absorbance was measured at 593 nm using a UV-1800 spectrophotometer (Shimadzu, Tokyo, Japan). All analyses were carried out in triplicate. Total antioxidant capacity was again expressed as Trolox equivalent per 100 g of dry weight (μmol TE/100 g DW).

### 3.5. Determination of Total Polyphenolic Content

Total polyphenols content was determined using Folin–Ciocalteu’s assay, originally described by Singelton & Rossi in 1965 [58]. Briefly, 0.1 mL of each extract was mixed with 3.8 mL of distilled water and 0.1 mL of the Folin–Ciocalteu reagent. After 3 min incubation in the dark, 1 mL of 10% (*w*/*v*) Na_2_CO_3_ solution was added to the mixture. The solution was then mixed vigorously and incubated at room temperature in the dark for 60 min. The absorbance was measured at 765 nm using a UV-1800 spectrophotometer (Shimadzu, Tokyo, Japan). Experiments were carried out in triplicate for each sample. The concentrations of total polyphenolic compounds in extracts were expressed as gallic acid equivalent per 100 g of dry weight (mg GAE/100 g DW).

### 3.6. Determination of Flavonoids Content

The determination of the content of flavonoids was performed using the method described by Chang et al. in 2002 [59] with modifications. An amount of 0.5 mL of each extract was separately mixed with 1.5 mL of 80% (*w*/*v*) ethanol solution, 0.1 mL of 10% (*w*/*v*) AlCl_3_ 6H_2_O, 0.1 mL of 1M CH_3_COONa and 2.8 mL of distilled water. After incubation in the dark at room temperature for 40 min, the absorbance was measured at 415 nm using a UV-1800 spectrophotometer (Shimadzu, Tokyo, Japan). Experiments were carried out in triplicate. Quercetin was used to prepare the standard calibration curve and the total flavonoids content was expressed as mg of quercetin per 100 g dry weight (mg quercetin/100 g DW).

### 3.7. UHPLC–DAD–ESI–MS/MS Analysis of Phenolic Compounds

Phenolic compounds in extracts obtained with different methods from *P. barbatus* roots were analyzed using the UHPLC–DAD–ESI–MS/MS method described by Oracz et al. in 2022 [60]. UHPLC analyses were performed using an UHPLC + Dionex UltiMate 3000 liquid chromatographic system consisting of a UHPLC pump, an autosampler, a column oven, a diode array detector with multiple wavelength (Thermo Fisher Scientific Inc., Waltham, MA, USA), and a Transcend™ TLX-2 multiplexed LC system equipped with a Q-Exactive Orbitrap mass spectrometer (Thermo Scientific, Hudson, NH, USA) using a heated electrospray ionization interface (HESI–II).

Separation was carried out using an Accucore™ C18 (2.1 × 150 mm, 2.6 μm particle size; Thermo Scientific, Pittsburgh, PA, USA column) with a two phase gradient system of 0.1% formic acid in water as mobile phase A, and acetonitrile as mobile phase B. The mobile-phase gradient used was: 0–8 min, 1–5% B; 8–15 min, 5–8% B; 15–20 min, 8–10% B; 20–25 min, 10–15% B; 25–35 min, 15–20% B; 35–40 min, 20–25% B; 40–50 min, 25–90% B; 50–53 min, 90% B; 53–58 min, 90–1% B; and finally, the initial conditions were held for 7 min for column re-equilibration. The flow rate of the mobile phase was 0.35 mL/min, and the column temperature was 30 °C. The injection volume was 10 μL. The chromatograms were recorded at three different wavelengths (i.e., 280, 320 and 365 nm).

The mass spectrometric conditions were as follows: capillary voltage, 4500 V; capillary temperature, 275 °C; heater gas temperature, 320 °C; sheath gas and auxiliary gas, nitrogen at flow of 35 and 15 (arbitrary units), respectively. Full scan mass spectra were acquired over a mass range from *m*/*z* 100 to 1500 in the negative ion mode. The MS/MS spectra were obtained in collision-induced dissociation (CID) mode with a normalized collision energy (NCE) of 20% [60].

The individual phenolic compounds were identified in extracts obtained with different methods from *P. barbatus* roots by comparing retention times, UV–vis absorbance spectra, full scan mass spectra, and MS/MS fragmentation patterns with their corresponding standards analyzed under identical conditions and previous literature reports [61] and searching the PubChem Substance and Compound (https://pubmed.ncbi.nlm.nih.gov, accessed on 21 October 2022) and ChemSrc (https://www.chemsrc.com/en/, accessed on 21 October 2022) databases.

Quantifications of the individual phenolic compounds in extracts were carried out using the external standard method, and the results were expressed as mg per g dry weight (mg/g DW).

### 3.8. Statistical Analysis

All the determinations were carried out in triplicate and the results presented below are the mean of 3 independent replications ± standard deviations (±SD). The significant differences among the means were estimated through Tukey’s HSD test. For all statistical analyses, *p* < 0.05 was considered as the statistical significance. The error bars in all figures correspond to the standard deviations.

## 4. Conclusions

Roots of *P. barbatus* are rich in polyphenolic compounds, especially flavonoids. The extraction efficiency was comparable for all methods and varies from 24.41% to 36.36%, while the average was 31%. Irrespective of the method and fraction of plant material, the lowest yield of the extraction process was obtained by the use of 80% ethanol as the solvent. Higher concentration of polyphenolic compounds, including flavonoids, was obtained from the finer fraction (≤0.5 mm). Similarly, the extracts obtained from the finer fraction were characterized by higher antioxidant capacity, which confirms that the degree of fragmentation of the plant material is of great importance for the efficiency of the extraction process. Comparing all the applied methods of extraction, UAE turned out to be the most effective in the extraction of polyphenols and antioxidants in general from dried and ground roots of *P. barbatus*. However, regardless of the extraction method, extending the extraction time does not significantly affect the efficiency of extraction, nor does it significantly affect the content of polyphenols, flavonoids and the total antioxidant capacity of the obtained extracts. Water extracts were characterized by the lowest antioxidant capacity as well as the lowest concentration of polyphenols and flavonoids compared to ethanol solutions. All ethanolic extracts were characterized by high antioxidant capacity. The 80% ethanol solution was noticed to be the best solvent for flavonoid extraction, while 40% and 60% ethanol solutions were adequate for the efficient extraction of the overall pool of polyphenols. A total of 15 phenolic compounds, belonging to the group of phenolic acids and their derivatives including quinic acid, potocatechuic acid, 4-hydroxybenzoic acid, 2-hydroxybenzoic acid, protocatechuic aldehyde, gentisic acid, 4-hydroxybenzoic acid *O*-hexoside, vanillic acid, caffeic acid, syringic acid, p-coumaric acid, ferulic acid, ellagic acid, hydroxygallic acid and rosmarinic acid were identified by UHPLC–DAD–ESI–MS/MS analysis. Rosmarinic acid was the dominant phenolic compound in almost all the extracts tested. RA concentration was several times higher in ethanol extracts than in aqueous ones, which additionally confirms the right choice of ethanol as a solvent to extract polyphenolic compounds from *P.barbatus* roots.

A review of the literature and conducted experiments indicate the possibility of using *P. barbatus* extracts rich in rosmarinic acid in dietary supplements or drugs used in the treatment of digestive disorders. In addition, extracts from the root of *P. barbatus* could be used as an ingredient of a dietary supplement or functional food products recommended as part of therapy in metabolic diseases. As forskolin stimulates lipolysis, supplements with P. barbatus extract may be effective as a support of a weight-loss diet. Due to the antimicrobial properties, *P. barbatus* extracts can be used in mouthwash preparations to prevent the development of microorganisms as well as in cosmetics. A new direction of research is the assessment of the properties of forskolin and other bioactive compounds present in *P. barbatus* extracts in the therapy of neurodegenerative diseases. The ability of some compounds present in *P. barbatus* extracts to inhibit the AchE enzyme combined with high antioxidant activity suggests that they may be a potential natural drug in the treatment of Alzheimer’s disease. Further experiments will be required to identify all the active molecules present in *P. barbatus* extracts and to understand their mechanism of action.

## Figures and Tables

**Table 1:** Yield of extracts obtained with different methods from *P. barbatus (Coleus forskohlii)* roots.

Fraction	Extraction Method	Solvent	Extraction Yield (%)
Fraction ≤ 0.5 mm	SWE 30	H_2_O	29.6 ± 0.7 ^a^
		40% ethanol	32.7 ± 0.7 ^c^
		60% ethanol	30.9 ± 0.5 ^b^
		80% ethanol	28.7 ± 0.5 ^a^
	SWE 60	H_2_O	32.7 ± 0.9 ^b^
		40% ethanol	32.2 ± 0.7 ^b^
		60% ethanol	33.9 ± 0.5 ^c^
		80% ethanol	28.4 ± 0.6 ^a^
	UAE 15	H_2_O	32.2 ± 0.9 ^c^
		40% ethanol	29.3 ± 0.6 ^a,b^
		60% ethanol	30.0 ± 0.3 ^b^
		80% ethanol	28.3 ± 0.5 ^a^
	UAE 30	H_2_O	31.2 ± 0.9 ^b^
		40% ethanol	32.9 ± 0.8 ^c^
		60% ethanol	31.4 ± 0.3 ^b^
		80% ethanol	28.7 ± 0.7 ^a^
	MAE	H_2_O	33.9 ± 0.9 ^c^
		40% ethanol	33.6 ± 0.3 ^c^
		60% ethanol	32.5 ± 0.7 ^b,c^
		80% ethanol	29.2 ± 0.7 ^a^
Fraction 0.5–1.25 mm	SWE 30	H_2_O	31.9 ± 0.9 ^c^
		40% ethanol	34.2 ± 0.6 ^d^
		60% ethanol	30.1 ± 0.8 ^b^
		80% ethanol	27.3 ± 0.9 ^a^
	SWE 60	H_2_O	31.5 ± 0.8 ^b^
		40% ethanol	31.4 ± 0.7 ^b^
		60% ethanol	31.9 ± 0.1 ^b^
		80% ethanol	27.7 ± 0.3 ^a^
	UAE 15	H_2_O	35.1 ± 0.8 ^c^
		40% ethanol	32.4 ± 0.7 ^b^
		60% ethanol	31.4 ± 0.6 ^b^
		80% ethanol	27.1 ± 0.3 ^a^
	UAE 30	H_2_O	33.0 ± 0.6 ^c^
		40% ethanol	32.0 ± 0.5 ^c^
		60% ethanol	30.5 ± 0.9 ^b^
		80% ethanol	27.2 ± 0.8 ^a^
	MAE	H_2_O	36.4 ± 0.8 ^d^
		40% ethanol	32.0 ± 0.5 ^c^
		60% ethanol	30.1 ± 0.7 ^b^
		80% ethanol	24.4 ± 0.8 ^a^

SWE—shaking water bath extraction; UAE—ultrasound-assisted extraction; MAE—microwave assisted extraction; 15, 30 or 60-time of extraction. Data are presented as mean ± SD of three replications. Different letters: a, b, c, d, indicate that samples are significantly different. The letter “a” marks the lowest value. Higher values are marked with consecutive letters of the alphabet. Different letters for each fraction and method in the same column indicate that samples are significantly different according to Tukey’s HSD test at *p* < 0.05.

**Table 2:** Phenolic composition of the extracts obtained with different methods from *P. barbatus (Coleus forskohlii*) roots (mg/g DW).

Extraction Method	Solvent	QA	PA	4-HBA	PAL	GA	HBA-hex	VA	CA	SA	p-CA	FA	2-HBA	EA	HGA	RA	TOTAL
SWE 30 Fraction <0.5	H_2_O	0.259 ± 0.032	0.376 ± 0.083	0.122 ± 0.017	0.073 ± 0.016	0.067 ± 0.014	0.015 ± 0.000	0.041 ± 0.000	0.237 ± 0.035	0.008 ± 0.000	0.008 ± 0.003	0.018 ± 0.000	0.025 ± 0.000	nd	0.035 ± 0.001	0.153 ± 0.007	1.437 ± 0.065
	40% EtOH	0.466 ± 0.113	0.418 ± 0.025	0.230 ± 0.025	0.123 ± 0.023	0.127 ± 0.036	0.027 ± 0.001	0.054 ± 0.000	0.169 ± 0.025	0.011 ± 0.001	0.008 ± 0.001	0.021 ± 0.000	nd	0.047 ± 0.003	0.148 ± 0.012	1.113 ± 0.028	2.962 ± 0.098
	60% EtOH	0.405 ± 0.061	0.428 ± 0.023	0.238 ± 0.035	0.122 ± 0.013	0.131 ± 0.013	0.027 ± 0.002	0.056 ± 0.001	0.176 ± 0.026	0.015 ± 0.003	0.008 ± 0.001	0.022 ± 0.000	0.025 ± 0.000	0.073 ± 0.001	0.157 ± 0.012	1.179 ± 0.021	3.062 ± 0.078
	80% EtOH	0.379 ± 0.033	0.401 ± 0.095	0.229 ± 0.016	0.124 ± 0.046	0.135 ± 0.026	0.026 ± 0.002	0.057 ± 0.002	0.174 ± 0.023	0.015 ± 0.004	0.008 ± 0.002	0.023 ± 0.001	0.022 ± 0.001	0.045 ± 0.002	0.145 ± 0.021	1.151 ± 0.011	2.934 ± 0.093
SWE 30 Fraction 0.5–1.25	H_2_O	0.415 ± 0.014	0.435 ± 0.083	0.154 ± 0.012	0.112 ± 0.013	0.104 ± 0.013	0.024 ± 0.001	0.057 ± 0.010	0.244 ± 0.035	0.011 ± 0.000	0.009 ± 0.001	0.018 ± 0.003	0.019 ± 0.001	0.047 ± 0.002	0.037 ± 0.009	0.214 ± 0.003	1.900 ± 0.057
	40% EtOH	0.499 ± 0.021	0.374 ± 0.123	0.213 ± 0.034	0.129 ± 0.024	0.128 ± 0.023	0.031 ± 0.005	0.058 ± 0.000	0.161 ± 0.032	0.011 ± 0.002	0.008 ± 0.003	0.020 ± 0.004	0.025 ± 0.000	0.036 ± 0.001	0.148 ± 0.008	1.126 ± 0.022	2.968 ± 0.062
	60% EtOH	0.523 ± 0.032	0.374 ± 0.065	0.238 ± 0.076	0.136 ± 0.037	0.135 ± 0.015	0.033 ± 0.000	0.065 ± 0.010	0.176 ± 0.016	0.012 ± 0.003	0.009 ± 0.000	0.022 ± 0.003	0.023 ± 0.002	0.038 ± 0.000	0.178 ± 0.016	1.196 ± 0.004	3.157 ± 0.060
	80% EtOH	0.548 ± 0.112	0.322 ± 0.023	0.200 ± 0.023	0.123 ± 0.013	0.128 ± 0.012	0.025 ± 0.000	0.058 ± 0.000	0.162 ± 0.012	0.011 ± 0.000	0.008 ± 0.000	0.021 ± 0.005	0.020 ± 0.002	0.033 ± 0.000	0.163 ± 0.024	1.066 ± 0.024	2.888 ± 0.078
SWE 60 Fraction <0.5	H_2_O	0.278 ± 0.033	0.425 ± 0.035	0.138 ± 0.044	0.086 ± 0.012	0.078 ± 0.019	0.023 ± 0.000	0.050 ± 0.002	0.286 ± 0.033	0.008 ± 0.000	0.009 ± 0.000	0.021 ± 0.002	0.019 ± 0.000	0.134 ± 0.000	0.043 ± 0.006	0.179 ± 0.021	1.777 ± 0.062
	40% EtOH	0.373 ± 0.024	0.412 ± 0.038	0.230 ± 0.016	0.125 ± 0.011	0.126 ± 0.028	0.036 ± 0.001	0.054 ± 0.008	0.169 ± 0.017	0.010 ± 0.000	0.008 ± 0.000	0.021 ± 0.000	0.023 ± 0.000	0.074 ± 0.012	0.143 ± 0.010	1.106 ± 0.034	2.910 ± 0.069
	60% EtOH	0.420 ± 0.125	0.444 ± 0.054	0.239 ± 0.012	0.129 ± 0.033	0.141 ± 0.015	0.030 ± 0.000	0.060 ± 0.007	0.192 ± 0.011	0.016 ± 0.004	0.008 ± 0.000	0.024 ± 0.005	0.035 ± 0.000	0.085 ± 0.014	0.169 ± 0.009	1.264 ± 0.072	3.256 ± 0.098
	80% EtOH	0.411 ± 0.052	0.441 ± 0.066	0.246 ± 0.049	0.134 ± 0.086	0.146 ± 0.023	0.025 ± 0.000	0.062 ± 0.001	0.194 ± 0.049	0.017 ± 0.005	0.0090 ± 0.001	0.024 ± 0.006	0.024 ± 0.001	0.054 ± 0.002	0.165 ± 0.027	1.242 ± 0.086	3.194 ± 0.083
SWE 60 Fraction 0.5–1.25	H_2_O	0.426 ± 0.093	0.435 ± 0.15	0.158 ± 0.010	0.125 ± 0.013	0.108 ± 0.047	0.033 ± 0.002	0.060 ± 0.000	0.221 ± 0.054	0.013 ± 0.001	0.049 ± 0.002	0.019 ± 0.000	0.019 ± 0.000	0.047 ± 0.000	0.029 ± 0.005	0.130 ± 0.012	1.873 ± 0.068
	40% EtOH	0.980 ± 0.123	0.353 ± 0.065	0.207 ± 0.084	0.122 ± 0.048	0.121 ± 0.014	0.029 ± 0.003	0.057 ± 0.000	0.155 ± 0.030	0.011 ± 0.001	0.007 ± 0.000	0.018 ± 0.001	nd	0.027 ± 0.000	0.153 ± 0.014	1.145 ± 0.045	3.386 ± 0.084
	60% EtOH	0.505 ± 0.032	0.336 ± 0.023	0.226 ± 0.056	0.131 ± 0.016	0.133 ± 0.006	0.056 ± 0.002	0.063 ± 0.004	0.165 ± 0.014	0.018 ± 0.002	0.009 ± 0.000	0.020 ± 0.001	0.021 ± 0.000	0.033 ± 0.000	0.178 ± 0.014	1.065 ± 0.090	2.959 ± 0.079
	80% EtOH	0.543 ± 0.071	0.328 ± 0.023	0.196 ± 0.034	0.117 ± 0.013	0.128 ± 0.013	0.022 ± 0.001	0.058 ± 0.000	0.161 ± 0.035	0.011 ± 0.004	0.008 ± 0.000	0.019 ± 0.000	0.018 ± 0.000	0.031 ± 0.000	0.159 ± 0.032	1.120 ± 0.128	2.919 ± 0.067
UAE 15 Fraction <0.5	H_2_O	0.353 ± 0.094	0.543 ± 0.093	0.167 ± 0.013	0.103 ± 0.044	0.094 ± 0.014	0.031 ± 0.000	0.059 ± 0.000	0.317 ± 0.032	0.014 ± 0.001	0.0114 ± 0.002	0.025 ± 0.001	0.027 ± 0.001	0.097 ± 0.001	0.055 ± 0.011	0.191 ± 0.033	2.087 ± 0.063
	40% EtOH	0.503 ± 0.035	0.445 ± 0.034	0.251 ± 0.030	0.153 ± 0.013	0.170 ± 0.024	0.029 ± 0.000	0.057 ± 0.007	0.179 ± 0.002	0.015 ± 0.002	0.0082 ± 0.000	0.024 ± 0.000	0.025 ± 0.002	0.125 ± 0.021	0.168 ± 0.012	1.214 ± 0.256	3.366 ± 0.075
	60% EtOH	0.414 ± 0.153	0.431 ± 0.162	0.240 ± 0.084	0.125 ± 0.015	0.135 ± 0.046	0.026 ± 0.000	0.057 ± 0.018	0.180 ± 0.012	0.017 ± 0.004	0.009 ± 0.000	0.022 ± 0.003	0.024 ± 0.000	0.114 ± 0.031	0.168 ± 0.013	1.186 ± 0.313	3.148 ± 0.078
	80% EtOH	0.476 ± 0.049	0.392 ± 0.036	0.219 ± 0.016	0.119 ± 0.013	0.132 ± 0.011	0.022 ± 0.000	0.055 ± 0.017	0.172 ± 0.014	0.014 ± 0.001	0.008 ± 0.000	0.022 ± 0.000	0.021 ± 0.001	0.044 ± 0.001	0.142 ± 0.035	1.101 ± 0.084	2.939 ± 0.063
UAE 15 Fraction 0.5–1.25	H_2_O	0.473 ± 0.176	0.480 ± 0.122	0.159 ± 0.049	0.129 ± 0.048	0.112 ± 0.011	0.033 ± 0.000	0.058 ± 0.007	0.240 ± 0.024	0.012 ± 0.002	0.010 ± 0.000	0.019 ± 0.000	0.024 ± 0.002	0.052 ± 0.001	0.040 ± 0.015	0.219 ± 0.025	2.060 ± 0.065
	40% EtOH	0.469 ± 0.0951	0.350 ± 0.064	0.207 ± 0.097	0.131 ± 0.029	0.124 ± 0.012	0.027 ± 0.000	0.058 ± 0.019	0.149 ± 0.014	0.011 ± 0.000	0.008 ± 0.000	0.019 ± 0.001	0.021 ± 0.000	0.060 ± 0.002	0.149 ± 0.015	1.064 ± 0.073	2.847 ± 0.069
	60% EtOH	0.513 ± 0.0451	0.379 ± 0.074	0.197 ± 0.087	0.135 ± 0.017	0.129 ± 0.021	0.024 ± 0.000	0.057 ± 0.009	0.165 ± 0.011	0.012 ± 0.006	0.008 ± 0.001	0.021 ± 0.000	0.022 ± 0.000	0.035 ± 0.001	0.167 ± 0.029	1.181 ± 0.163	3.045 ± 0.075
	80% EtOH	0.457 ± 0.133	0.322 ± 0.082	0.192 ± 0.028	0.124 ± 0.025	0.124 ± 0.009	0.045 ± 0.001	0.055 ± 0.000	0.158 ± 0.021	0.016 ± 0.007	0.008 ± 0.000	0.019 ± 0.001	nd	0.030 ± 0.00	0.136 ± 0.089	0.969 ± 0.033	2.655 ± 0.085
UAE 30 Fraction <0.5	H_2_O	0.275 ± 0.021	0.398 ± 0.045	0.135 ± 0.015	0.078 ± 0.017	0.074 ± 0.012	0.018 ± 0.002	0.044 ± 0.004	0.233 ± 0.026	0.008 ± 0.006	0.0085 ± 0.000	0.018 ± 0.002	0.016 ± 0.000	0.065 ± 0.002	0.041 ± 0.003	0.202 ± 0.025	1.614 ± 0.092
	40% EtOH	0.393 ± 0.064	0.409 ± 0.085	0.212 ± 0.049	0.118 ± 0.015	0.124 ± 0.012	0.025 ± 0.000	0.054 ± 0.0014	0.169 ± 0.016	0.010 ± 0.001	0.0076 ± 0.000	0.022 ± 0.001	0.023 ± 0.000	0.078 ± 0.004	0.148 ± 0.013	1.104 ± 0.085	2.897 ± 0.078
	60% EtOH	0.396 ± 0.063	0.417 ± 0.075	0.223 ± 0.026	0.122 ± 0.024	0.124 ± 0.002	0.046 ± 0.0130	0.055 ± 0.120	0.174 ± 0.024	0.014 ± 0.002	0.008 ± 0.002	0.022 ± 0.000	nd	0.079 ± 0.000	0.152 ± 0.064	1.108 ± 0.091	2.940 ± 0.072
	80% EtOH	0.381 ± 0.047	0.402 ± 0.042	0.227 ± 0.012	0.120 ± 0.046	0.131 ± 0.012	0.017 ± 0.000	0.056 ± 0.011	0.179 ± 0.079	0.017 ± 0.03	0.008 ± 0.000	0.022 ± 0.000	0.021 ± 0.000	0.084 ± 0.000	0.150 ± 0.032	1.084 ± 0.049	2.900 ± 0.089
UAE 30 Fraction 0.5–1.25	H_2_O	0.402 ± 0.074	0.501 ± 0.062	0.164 ± 0.014	0.129 ± 0.025	0.114 ± 0.002	0.021 ± 0.000	0.059 ± 0.000	0.256 ± 0.085	0.012 ± 0.001	0.010 ± 0.003	0.020 ± 0.002	0.020 ± 0.000	0.084 ± 0.001	0.039 ± 0.010	0.219 ± 0.020	2.051 ± 0.084
	40% EtOH	0.451 ± 0.094	0.352 ± 0.034	0.202 ± 0.016	0.125 ± 0.035	0.120 ± 0.007	0.024 ± 0.001	0.054 ± 0.000	0.145 ± 0.033	0.011 ± 0.000	0.007 ± 0.000	0.019 ± 0.001	nd	0.028 ± 0.000	0.154 ± 0.031	1.038 ± 0.004	2.730 ± 0.069
	60% EtOH	0.496 ± 0.117	0.350 ± 0.013	0.217 ± 0.014	0.129 ± 0.032	0.129 ± 0.031	0.022 ± 0.000	0.058 ± 0.007	0.163 ± 0.004	0.012 ± 0.000	0.009 ± 0.002	0.021 ± 0.002	0.021 ± 0.000	0.025 ± 0.000	0.161 ± 0.012	1.129 ± 0.144	2.942 ± 0.075
	80% EtOH	0.566 ± 0.053	0.326 ± 0.063	0.203 ± 0.014	0.125 ± 0.021	0.126 ± 0.002	0.043 ± 0.000	0.058 ± 0.006	0.165 ± 0.048	0.012 ± 0.003	0.008 ± 0.000	0.022 ± 0.000	0.026 ± 0.002	0.026 ± 0.000	0.158 ± 0.087	1.126 ± 0.079	2.990 ± 0.082
MAE Fraction <0.5	H_2_O	0.278 ± 0.037	0.387 ± 0.014	0.132 ± 0.014	0.077 ± 0.037	0.075 ± 0.011	0.014 ± 0.000	0.044 ± 0.005	0.201 ± 0.024	0.011 ± 0.001	0.008 ± 0.000	0.017 ± 0.000	0.019 ± 0.000	0.069 ± 0.003	0.037 ± 0.009	0.195 ± 0.041	1.564 ± 0.085
	40% EtOH	0.414 ± 0.033	0.421 ± 0.093	0.216 ± 0.062	0.124 ± 0.871	0.125 ± 0.002	0.019 ± 0.002	0.052 ± 0.009	0.156 ± 0.053	0.015 ± 0.005	0.008 ± 0.001	0.021 ± 0.002	nd	0.067 ± 0.000	0.149 ± 0.013	1.072 ± 0.086	2.859 ± 0.089
	60% EtOH	0.398 ± 0.063	0.395 ± 0.062	0.221 ± 0.032	0.123 ± 0.021	0.131 ± 0.037	0.018 ± 0.001	0.054 ± 0.008	0.169 ± 0.055	0.013 ± 0.002	0.008 ± 0.000	0.022 ± 0.000	0.023 ± 0.001	0.072 ± 0.002	0.155 ± 0.005	1.134 ± 0.099	2.936 ± 0.078
	80% EtOH	0.485 ± 0.125	0.398 ± 0.072	0.218 ± 0.021	0.125 ± 0.0784	0.133 ± 0.002	0.033 ± 0.004	0.056 ± 0.002	0.175 ± 0.034	0.017 ± 0.000	0.009 ± 0.000	0.023 ± 0.000	0.018 ± 0.000	0.038 ± 0.000	0.145 ± 0.001	1.103 ± 0.069	2.976 ± 0.093
MAE Fraction 0.5–1.25	H_2_O	0.432 ± 0.043	0.468 ± 0.021	0.133 ± 0.051	0.122 ± 0.022	0.099 ± 0.009	0.016 ± 0.000	0.056 ± 0.001	0.218 ± 0.068	0.014 ± 0.004	0.007 ± 0.000	0.018 ± 0.000	0.023 ± 0.000	0.071 ± 0.002	0.031 ± 0.009	0.132 ± 0.071	1.841 ± 0.078
	40% EtOH	0.738 ± 0.025	0.352 ± 0.046	0.203 ± 0.016	0.129 ± 0.037	0.120 ± 0.002	0.044 ± 0.000	0.056 ± 0.005	0.147 ± 0.013	0.016 ± 0.000	0.008 ± 0.000	0.018 ± 0.000	nd	0.075 ± 0.005	0.146 ± 0.031	1.016 ± 0.131	3.068 ± 0.094
	60% EtOH	0.470 ± 0.024	0.342 ± 0.068	0.198 ± 0.043	0.124 ± 0.0.029	0.127 ± 0.014	0.040 ± 0.000	0.057 ± 0.007	0.156 ± 0.031	0.012 ± 0.001	0.008 ± 0.000	0.019 ± 0.000	0.021 ± 0.000	0.024 ± 0.000	0.162 ± 0.008	1.078 ± 0.071	2.839 ± 0.077
	80% EtOH	0.395 ± 0.085	0.262 ± 0.017	0.171 ± 0.034	0.108 ± 0.0.011	0.114 ± 0.014	nd	0.051 ± 0.000	0.136 ± 0.009	0.014 ± 0.000	0.007 ± 0.000	0.017 ± 0.000	0.012 ± 0.000	0.058 ± 0.009	0.125 ± 0.097	0.806 ± 0.032	2.277 ± 0.085

Results are presented as the mean ± SD from triplicate determinations. Nd—not detected; SWE—shaking water bath extraction; UAE—ultrasound-assisted extraction; MAE—microwave assisted extraction; 15, 30 or 60-time of extraction. QA, Quinic acid; PA, Protocatechuic acid; 4-HBA, 4-Hydroxybenzoic acid; PAL, Protocatechuic aldehyde; GA, Gentisic acid; HBA-hex, 4-Hydroxybenzoic acid O-hexoside; VA, Vanillic acid; CA, Caffeic acid; SA, Syringic acid; p-CA, p-Coumaric acid; FA, Ferulic acid; 2-HBA, 2-Hydroxybenzoic acid; EA, Ellagic acid; HGA, Hydroxygallic acid; RA, Rosmarinic acid.

**Table 3:** Variants of the extraction of bioactive compounds from *P. barbatus* (*Coleus forskohlii*) roots with extraction parameters.

Extraction Method	Solvent	Time of Extraction
Shaking water bath extraction (SWA)	H_2_O	30 min
	40% ethanol	30 min
	60% ethanol	30 min
	80% ethanol	30 min
	H_2_O	60 min
	40% ethanol	60 min
	60% ethanol	60 min
	80% ethanol	60 min
Ultrasound-assisted extraction (UAE)	H_2_O	15 min
	40% ethanol	15 min
	60% ethanol	15 min
	80% ethanol	15 min
	H_2_O	30 min
	40% ethanol	30 min
	60% ethanol	30 min
	80% ethanol	30 min
Microwave assisted extraction (MAE)	H_2_O	10 s
	40% ethanol	9 s
	60% ethanol	8 s
	80% ethanol	7 s

## Data Availability

Data will be made available on request.

## Sample Availability

Samples of the compounds are available from the authors.
